# From Prevention to Therapy: A Roadmap of Nanotechnologies to Stay Ahead of Future Pandemics

**DOI:** 10.1021/acsnano.2c04148

**Published:** 2022-07-06

**Authors:** Sutapa Chandra, Tony Hu

**Affiliations:** † Center for Cellular and Molecular Diagnostics, Tulane University School of Medicine, New Orleans, Louisiana 70112, United States; ‡ Department of Biochemistry and Molecular Biology, Tulane University School of Medicine, New Orleans, Louisiana 70112, United States

**Keywords:** nanotechnology, virucides, diagnostics, therapy, prevention, vaccines, SARS-CoV-2

## Abstract

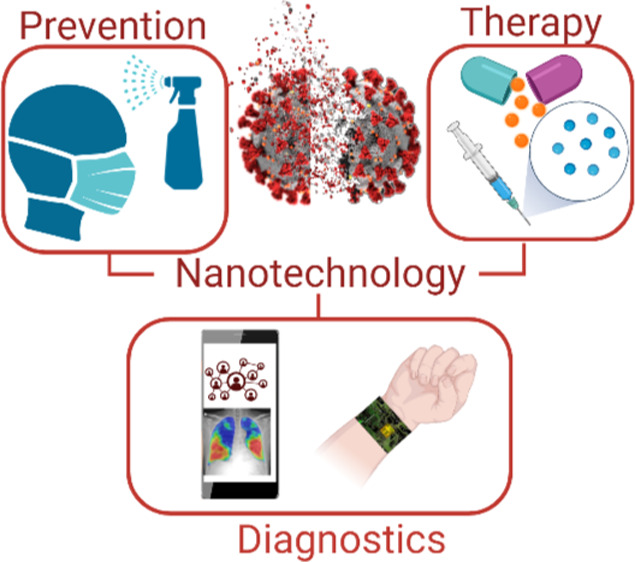

Several recent viral outbreaks, culminating in the COVID-19 pandemic, have illustrated the need for comprehensive improvement in the detection, control, and treatment of emerging viruses that exhibit the potential to cause epidemics. Nanotechnology approaches have the potential to make major contributions in all these areas. This perspective is intended to outline how nanotechnology can be employed to improve upon respiratory disease detection and containment measures, and therapeutics, with a particular emphasis on applications that can address key areas, including home diagnostics, contact tracing, and the evaluation of durability of vaccine protection over time and against future variants. Nanotechnology offers potent tools to address these needs, but further research is required to validate these applications to address needs of future epidemics.

Global efforts to eradicate severe acute respiratory syndrome coronavirus 2 (SARS-CoV-2) have led to acceptance that this virus has or will become endemic in the global population. Learning mistakes made during early SARS-CoV-2 diagnosis and containment efforts is critical to prevent or contain future outbreaks of SARS-CoV-2 variants of concern or emerging pathogens capable of producing pandemics. One of these key lessons had been the critical need for simple, rapid, and inexpensive point-of-care diagnostics that can accurately detect forthcoming cases to prevent large outbreaks. Vaccines and antibody therapeutics can provide a more long-term solution given their development and validation time, although their effectiveness can be diminished by variants that arise during ongoing transmission and by the progressive decline in protective immunity following vaccination. Broad spectrum vaccines that target multiple SARS-CoV-2 variants of concern (VOCs) are therefore of great interest for their potential to produce enduring protection against emerging variants. Most current vaccines target the spike protein of WuhanHu-1 strain,^1^ which can reduce their ability to produce immune responses that can rapidly respond to current and future SARS-CoV-2 VOCs. However, the use of bivalent vaccine boosters that also contain sequences from the SARS-CoV-2 Omicron VOC is also under evaluation.^2,3^ Future SARS-CoV-2 vaccines may require seasonal updates in response to predictions made using epidemiologic data concerning emerging VOCs,^4^ similar to the influenza vaccine production. However, there is significant interest for multivalent vaccines that display spike proteins from a pool of VOCs to enhance potential protective coverage of a predictive vaccine, and the development of vaccines that can target conserved regions that can offer protection against a broad array of potential VOCs. Broad spectrum therapeutics are also of considerable interest, and studies examining the efficacy of oral antiviral drugs to reduce severe illness by SARS-CoV-2 variants when taken soon after symptom onset and to prevent or attenuate long COVID pathology are now underway. Further developments on all these topics are required to permit the world to “live with SARS-CoV-2” and deal with future pathogens that have the potential to cause pandemics. This perspective will employ SARS-CoV-2 as an example to discuss what measures may be useful in addressing these issues.

What lies ahead? Countries around the world are easing restrictions and stepping toward economic recovery, but while this is inevitable at some point, it risks another wave of infection that may further damage already strained economic and healthcare infrastructure. To minimize the potential for and extent of such events, additional worldwide effort should be focused on the development of smart strategies to allow the safe resumption of daily activities by developing more advanced means to rapidly detect future outbreaks and variants and better means of treatment. Specifically, nanotechnology approaches provide multiple opportunities to improve critical means of infection prevention and detection, including personal protection measures, environmental sanitation approaches, and rapid diagnostic tests, among others (Figure 1). For example, scalable hand-held or wearable electronics that can rapidly detect infection could be an essential component of efforts to rapidly detect and detect impending infectious disease cases that have the potential to cause future pandemics. Nanotechnology may play a critical role in such devices due to its current influence on point-of-care diagnostics and potential to offer a rich palette of modular biosensor designs useful in user-friendly, inexpensive, and portable diagnostic devices.

**Figure 1:**
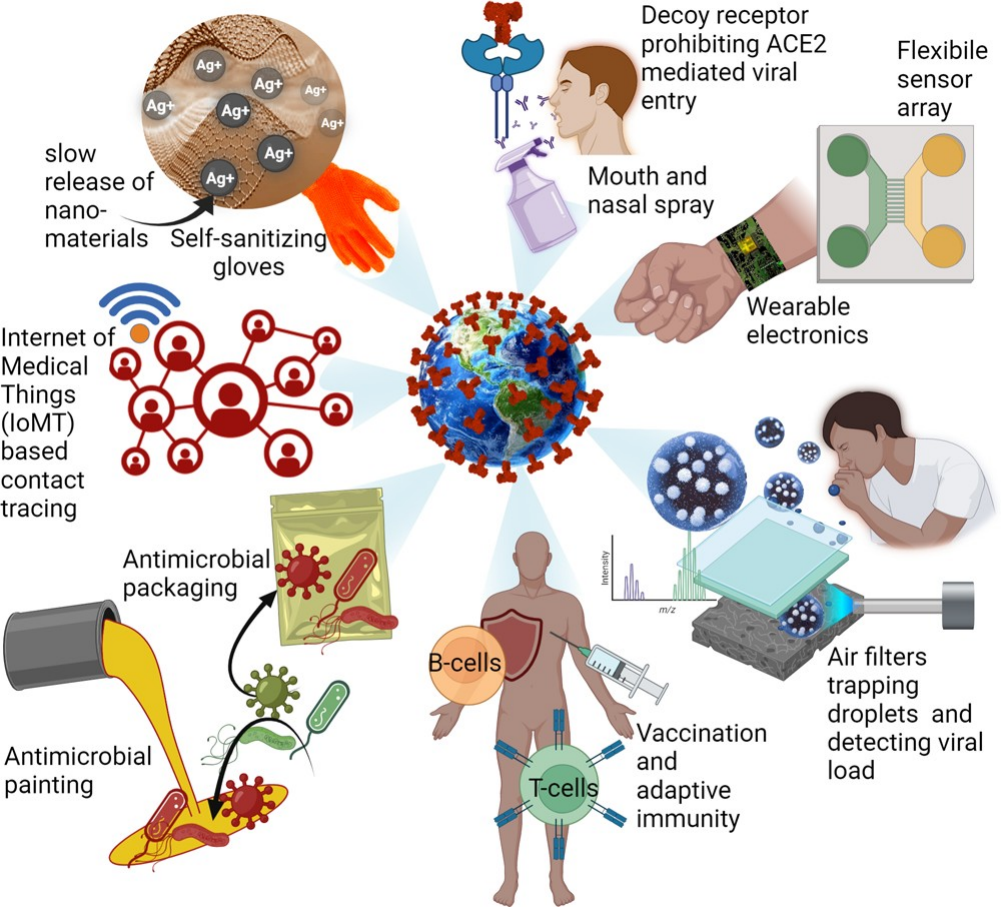
Schematic depicting potential uses of nanomaterials in the prevention, diagnosis, and treatment of viral infections to prevent future epidemics and pandemics. Figure created with BioRender.com

## Personal Protection Measures

SARS-CoV-2 transmission predominantly occurs by inhalation of aerosols and droplets generated by coughing, sneezing, or talking, with surface transmission appearing to play a modest role.^5^ Measures employed to reduce respiratory transmission, such as facemasks, have played a key role in reducing transmission, but extended use of facemasks in day-to-day life can have drawbacks, including physical discomfort, that can reduce compliance even in individuals willing to adhere to their use. Facemasks intended for protection against respiratory pathogens should have excellent filtration efficacy and be comfortable when worn for extended time periods. However, current N95 and surgical facemasks lack self-sterilizing properties and are thus prone to accumulate pathogens during the course of prolonged use, leading to increased risk of exposure. One recent study found that facemasks coated with a graphene nanolayer could block interactions with small droplets, which tended to bounce off this superhydrophobic nanolayer.^6^ Further, exposing this graphene nanolayer surface to direct sunlight for 1 min could raise its temperature to >80 °C, allowing its photothermal sterilization to permit extended and repeated mask usage without the need for harsh chemical sterilization procedures that could damage their filtration performance. This study also found that conjugation of silver nanoparticles within the graphene layers improved the disinfectant characteristics of the facemasks. The addition of thin copper nanowire metal–organic framework core–shell structures^7^ or copper oxide nanoparticles into facemask designs was also found to be a promising means for incorporating antiviral activity.^8,9^ Another key limitation of the traditional facemask is the thermal discomfort or skin damage caused by constant use. The thermal characteristics of a facemask primarily depend on fiber thickness, which influences air filtration and permeability. Studies indicate that facemasks that use nanofibers can reduce the respiratory effort required during normal air filtration, while still protecting protect against small particles (<50 nm).^10^ Nanoporous polyethylene nanofibers can provide filtration efficiencies superior to those of traditional facemasks material, while their infrared transparency allows improved radiative cooling to reduce thermal discomfort.^10,11^ Thus, nanomaterials have potential to improve the performance of traditional mask designs by incorporating self-sanitizing and antiviral/antimicrobial activities, reducing their insulating properties to increase comfort, and improving their particulate filtration properties. Several external parameters can affect the filtration efficacy and comfort of a facemask, including the environmental temperature and humidity, which can promote overheating and condensation to promote the growth and survival of microorganisms on its filtration materials, and elevation, which can further restrict air intake. Facemask design and composition can also affect the trade-off between filtration efficiency and air intake, but the use of nanomaterials can improve the filtration properties of current facemask designs. For example, nanomaterials can be incorporated into textiles to modify the fibers used in mask construction and improve their filtration efficiency by altering their electrostatic properties or other parameters. Several nanomaterials with antiviral activities, including those that employ silver, copper oxide, and carbon nanoparticles, have been successfully embedded into textiles used in facemasks.^12^ Additional nanomaterials properties, such as their relative hygroscopic and thermal properties, may also permit the design changes to produce more comfortable masks that still exhibit excellent filtration properties.

Hand sanitization is another essential personal protective measure that has become an inseparable part of personal safety in public settings where it is often not feasible to wash one’s hands with soap and water after exposures to high contact surfaces, such as public transport, shopping venues, and large public events. However, effective use of hand sanitizers requires either contactless application or sanitization of individuals containers during use to prevent possible recontamination from their surfaces. The Centers for Disease Control and Prevention recommends that such hand sanitizers contain at least 60% alcohol, but widespread and frequent use of such hand sanitizers can entail risks. For example, the Food and Drug Administration found 50 adverse events attributed to the use of alcohol-based sanitizers shortly after the start of the COVID-19 in March 2020.^13^ Another study conducted between January 2018 and December 2020 by the U.S. Poison Control Center identified 299 adverse events caused by abundant exposure to hand sanitizers, primarily headache and dizziness attributed to vapors from these products.^13^ Disposable medical gloves are an alternative for those who cannot tolerate hand sanitizers but cannot be readily sterilized and can thus transfer contamination, can be difficult to wear for extended periods, must be removed carefully to avoid hand contamination, are intended for single use and can be nonbiodegradable (vinyl or nitrile gloves) and thus present a disposal problem, or can have risks of contact dermatitis and even more severe allergic reactions in some individuals (latex gloves). These issues could, however, potentially be addressed by the use of self-sanitizing biodegradable gloves. For example, nanofibrous mesh layers that contain metal nanoparticles (e.g., copper and silver) with antiviral properties could be incorporated into the outer surface of a biodegradable glove design to reduce their surface contamination, reducing the potential for wearer exposure and secondary transfer of this contamination to other surfaces. The nanofibrous mesh structures of these gloves could also provide ventilation to reduce sweat accumulation inside the gloves, a common issue for latex and plastic gloves used in bioprotective applications.

## Environmental Sanitation

Many viruses, including SARS-CoV-2, can survive on surfaces for extended periods under favorable conditions, raising the potential for “supply chain contagion” where rapid national and international transport of contaminated packages could serve as a vector for distant outbreaks. Surface-based transmission events play a crucial role in the spread of several human viruses, including porcine epidemic diarrhea virus, the middle east respiratory syndrome (MERS) coronavirus, and endemic human coronaviruses 229E and OC43.^14^ Virus viability on a contaminated surface depends on multiple factors, including the surface type (porous or nonporous), virus structure (enveloped or nonenveloped), and temperature and humidity conditions. Recent studies indicate that SARS-CoV-2 viability is greater on plastic or stainless-steel surfaces (up to 72 h) than copper or cardboard surfaces (3–4 h),^15^ with greater virus viability and stability detected on nonporous versus porous surfaces.^16^ Many researchers have investigated viability by virus type and stain and found that surface survival times for enveloped viruses, including influenza virus, parainfluenza virus, and corona virus can vary from hours to days, whereas nonenveloped viruses, such as astroviruses and rotaviruses, can survive on surfaces for up to a few months.^14^

Ideal disinfectants should cover the entire antimicrobial spectrum, exhibit rapid activity without toxic effects on the environment or human health, be cost-effective, biodegradable, and compatible with a broad array of potential decontamination surface types. Alcohol-based disinfectants played a key role during the COVID-19 pandemic, but the virucidal efficacy of alcohol disinfectants varies with their alcohol concentration, contact time, and the targeted virus type. For example, isopropyl alcohol is effective only for enveloped viruses (e.g., herpesviruses, hepatitis viruses, SARS-CoV, etc.) and lacks effective virucidal activity against nonenveloped viruses, including adenoviruses and rhinoviruses that are responsible for common human infections.^17^ Frequent usage of concentrated alcohol solutions used in disinfectants also can damage many common surfaces, including plastics, rubber, and many wood finishes. New materials with broader virucidal activities and fewer downsides are thus highly desirable. Recent studies indicate that single-walled carbon nanotubes can be used in a spray disinfectant, since their photothermal activity could increase the temperature of treated surfaces to 90 °C when exposed to direct solar illumination for effective virucidal activity.^18^ Spraying these single-walled carbon nanotubes onto a polypropylene surface also formed nanospike structures that exhibited super hydrophobicity when exposed to various biological fluids to reduce surface contamination. A spray containing the trifunctional polymer poly(DMA-PEGMA-QA) has also been reported to provide at least 24 h of protection against viral contamination by forming a nanoscale coating layer when applied to the fabric of personal protective equipment.^19^ Biocompatibility tests found that poly(DMA-PEGMA-QA) did not induce any discernible toxicity in vitro or in vivo. Studies have also reported that low toxicity alginate-based nanocomposites can inhibit a wide spectrum of viruses, including the human immunodeficiency virus and the hepatitis A, B, and C, herpes, rabies, rubella, and polio viruses.^20^ Metal oxide nanomaterials, including a zinc oxide nanospray, are also effective as antiviral surface disinfectant agents,^21^ although care may need to be taken to balance the virucidal activity against potential cytotoxic effects. More research is therefore warranted to evaluate the potential utility of nanomaterial-based disinfectant sprays against the spectrum of virus types known to cause human disease. This potential for surface transmission raised questions about the safety of transported goods at the consumer level and changed the consumer-merchandise landscape early in the SARS-CoV-2 pandemic.^22,23^ Surface contamination of packages has not proven to be an issue for SARS-CoV-2 or other pathogens to date, but the incorporation of antiviral coatings or films on packaging materials could be used to reduce the potential for viral contamination, particularly when handling materials that may be more prone to such contamination. Hybrid nanocomposites containing copper,^7,24,25^ zinc, graphene,^26^ and silver^27,28^ have antiviral activities and could be incorporated into packaging materials or other materials used on high contact surfaces. Such nanocomposites can also be added to paints and varnishes to produce antiviral coatings capable of continuously decontaminating walls and other potential contract surfaces in public spaces, including airports, bus and train stations, public vehicles, and grocery stores, shopping malls, theaters, and healthcare units. Large-scale deployment of such antiviral coatings in paint and other materials thus represents another key approach for nonspecific reduction of viral surface contamination, which requires additional research to determine its best applications and potential utility to attenuate viral infections, including current and emerging viruses capable of major disease outbreaks.

Disinfectant sprays are commonly used to sanitize high contact surfaces in public areas, including public transport, healthcare facilities, as well as households. However, routine and/or excessive use of common disinfectant chemicals (e.g., alcohol, bleach, hydrogen peroxides, and several ammonium compounds) may have adverse effects on human health and the environment.^29^ There is thus a need for less hazardous disinfectants, which has led to research on the development of biocompatible disinfectants, including solutions that contain biocompatible metal nanoparticles, which could provide a safe alternative to disinfectant chemicals in current use. Nanomaterials are now in broad use in different biological applications, but concern should still be applied for their use in human health applications and the potential impact of such applications on the environment. Nanomaterial toxicity effects are mediated by their size, charge, and composition. Studies are still underway to investigate how physicochemical features of different nanomaterials influence toxicity.^30^ More research is therefore required to study toxic nanomaterial effects that could result from changes in their dimension, structure, surface area, functionality, constituent, source, and exposure dose before deploying them as a disinfectant spray.

## Diagnostic Approaches

The demand for and rapid expansion of decentralized and home-based testing technologies has been a dramatic effect of the COVID-19 pandemic. This demand has led to the development of simple diagnostic assays that use inexpensive portable or disposable sample-in-result-out readout devices. Such devices can eliminate the need to transport clinical samples to a centralized lab and eliminate the need for trained personnel and specialized equipment that delay results and can increase costs.

This development has produced both advantages and challenges, but greatly increased test capacity and sample-to-answer times at the cost of reduced testing accuracy and the loss of epidemiologic data that would normally be reported by testing laboratories. Nanomaterial applications can be employed to address the first of these drawbacks, and it should be possible to address the second with smartphone-based reporting apps or other approaches, although these still entail potential difficulties with user compliance and data security.

Several studies have evaluated nanomaterials for electroactive functional properties required to obtain the signal amplification required to sensitively detect the target virus across a wide concentration range in complex biological specimens including saliva and nasal tissue specimens employed by current SARS-CoV-2 diagnostics.^31−35^ An epidemiological study conducted early in the pandemic estimated that 80% of SARS-CoV-2 in the analyzed population could be attributed to virus transmission by an undiagnosed SARS-CoV-2 infected population.^36^ Thus, early detection measures can play a crucial role in disease control by rapidly identifying these infected individuals and implementing measures to reduce their potential for virus transmission. Reverse transcription polymerase chain reaction (RT-PCR) is the gold standard for SARS-CoV-2 RNA detection, but can be time-consuming since assays are usually collected and analyzed in batches in clinical laboratories that have the equipment and trained personnel required to perform these assays. It can thus require more than 24 h to obtain a test result after sample collection, providing a significant window for SARS-CoV-2 transmission from individuals with asymptomatic infections. Nanomaterial solutions have the potential to address this testing bottleneck to allow assays to be performed at sites lacking these resources to increase testing capacity and thus avoid shipping and processing bottlenecks that increase sample-to-answer times. For example, a plasmonic nanomaterial that rapidly and efficiently converts light to heat has been used to develop a simple photonic PCR device that permits ultrarapid template amplification and real-time analysis (40 PCR cycles in ∼5 min) with ∼90% yield, using an on-chip format ideal for point-of-care diagnosis at resource limited settings.^37^ Nanomaterial devices can also simplify the extraction of nucleic acids from biofluid, which is typically required from most conventional PCR assays, but can be a complex process that requires trained personnel and reagents and equipment that may be difficult to obtain at sites with limited resources. For example, single-walled carbon nanotubes can permit direct recovery of nucleic acid from complex biological samples using a single-tube isolation procedure that does not require multiple steps, expensive supplies or equipment, or substantial technical expertise.^38^ Nanodroplets loaded with target-specific Cas13a/CRISPR RNA complexes and quenched fluorescent probes have also been used to detect target RNAs in a digital droplet assay format, avoiding the need for RT-PCR,^39^ although this approach is not suitable for high-throughput analyses or use in resource limited settings. Nanomaterials have also made significant contributions to rapid serodiagnosis approaches. For example, a lateral flow immunoassay that uses fluorescent dye-loaded nanoparticles is reported to detect SARS-CoV-2-specific immunoglobulin G in most of the tested subjects within 1 day after symptoms onset^40^ and thus has a strong potential for rapid point-of-care diagnosis. Nanoparticles functionalized with virus-specific epitopes can also be used in seroprevalence surveys to identify individuals with late-stage or past SARS-CoV-2 infections, where positive cases are detected by a colorimetric change induced by nanoparticle aggregation upon cross-linking by these specific antibodies.^41^ A similar approach has also been employed for direct detection of SARS-CoV-2 RNA from processed patient specimens to permit rapid visual detection of positive samples.^42^ In both cases, this approach eliminates the need for sophisticated detection instruments to facilitate their use in point-of-care settings. Such nanomaterial sensors should also be readily incorporated into lab-on-a-chip devices employed in at-home testing kits. Future sensors should ideally also evaluate alternate sample types since reliance on upper respiratory tract specimens may miss respiratory virus infections in the lower respiratory tract that are not detected by these specimens due to limited or transient infection of these regions during an active infection. Diagnosis of these infections may require more invasive specimens (e.g., tracheal aspirate, bronchoalveolar lavage, or bronchial brush) or expensive computed tomography scans that can have poor diagnostic specificity. Lab-on-a-chip designs that couple the optical and chemical properties of nanomaterials to biological receptors for target detection have already been reported for diverse biosensing applications.^43−48^ However, even with advances in sensitivity and specificity, several concerns still need to be addressed before such devices can be used in large-scale applications. One such concern is that disposable readout devices and batteries would generate a substantial amount of electronic waste, although this could be offset if these devices could be made modular to accept small test chips and employ rechargeable batteries. Ideally, these devices should also be able to communicate their results in a format that permits their simple and secure transmission to a central epidemiologic data repository that can be used to monitor potential outbreaks. These concerns, and battery weight, also apply to wearable sensors and may be particularly relevant in poor in remote or resource limited settings. More attention thus needs to be paid to develop sensor platforms with small replaceable sensor chips and rechargeable batteries or other power supplies. Power generation approaches that employ small photovoltaic panels, generators that capture mechanical energy from small body movements, or other forms of renewable power could serve this purpose.

Wearable lab-on-a-chip devices could have significant clinical utility if they can rapidly detect early signs of infection to prevent the spread of infection and improve contact tracing and/or identify symptoms associated with the development of severe disease to allow rapid intervention that could minimize disease pathology and improve patient outcomes. Such wearable sensors may need direct physical contact with the skin through electroactive thin film sensors to monitor some key biometric parameters.^49−51^ Flexible electronics that use biocompatible nanomesh structures derived from polymeric nanomaterials could be adopted to develop flexible and gas permeable skin-contact sensors suitable for extended use in these applications. Integrating artificial intelligence (AI) into an Internet of medical things (IOMT) lab-on-a-chip device would be an incredibly powerful strategy to develop an extensive health surveillance framework. Such devices could allow real-time monitoring for changes in heart rate, blood oxygenation level, resting body temperature, or other parameters that could potentially be used as a suggestive indicator for asymptomatic, presymptomatic, or mild infections and provide a wealth of valuable epidemiologic information. Similarly, other wearable devices designed to constantly monitor for changes in blood oxygen levels, respiratory effort, changes in voice pitch, coughing, or other respiratory parameters in sick individuals and/or those at-risk for severe disease could be useful early indicators to seek rapid medical care. IOMT diagnostic devices could be used to detect new outbreaks, identify individuals who should be tested for infection, and assist in contact tracing and quarantine confirmation by transmitting real-time remote data and location signals remotely in several locations. Thus, AI-IOMT-assisted lab-on-a-chip devices appear likely to be one important pillar of next-generation diagnostic devices needed to avoid or reduce the extent and duration of future lockdowns or guide the reopening of business and social activities in the wake of such events.

The SARS-CoV-2 pandemic has also illustrated the need for assays that can accurately measure specific immune responses generated in response to vaccination or infection to predict the likelihood of protection from infection, severe infection, or death upon subsequent exposure. A better understanding of how these immune responses change over time and respond to future variants will be essential in allowing public health officials to make rapid and informed decisions on vaccine efficacy and dosing strategies and other policy measures. However, current methods have limited utility for monitoring the adaptive immune responses regulated by effector and memory B-cells and T-cells that are responsible for durable specific immunity after vaccination or exposure to a viral pathogen and can remain protective against SARS-CoV-2 infection after protective neutralizing antibodies decline below protective levels.^52^ Nanotechnology approaches have the potential to streamline current B- and T-cell response assays, which are not suitable for high-throughput use to monitor protective immunity at scale, to identify the decline in vaccine efficacy against future variants or to better evaluate the need for specific populations or individuals to receive a vaccine booster dose to achieve protective immunity.

## Therapeutic Approaches

**Figure 2:**
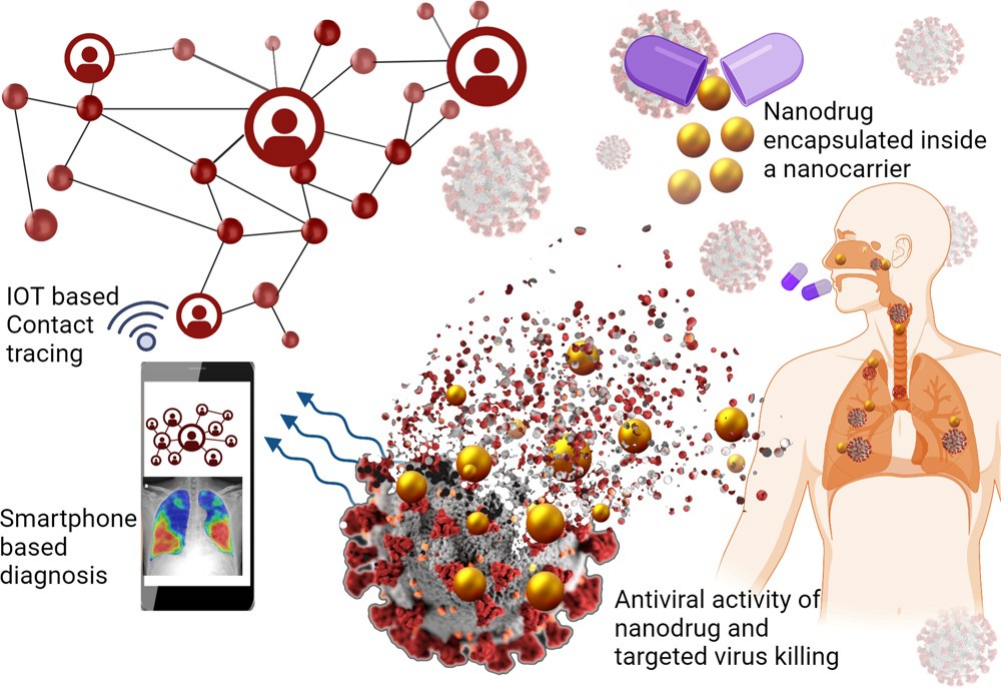
Schematic of the use of nanomaterials in nanocarriers, nanodecoys, and nanovaccines to enhance disease treatment or prevention approaches. Figure created with BioRender.com.

Nanomaterials may also have utility in three distinct types of therapeutic applications: nanocarriers that can permit targeted drug delivery, nanodecoys that can adsorb virus particles, or pro-inflammatory factors to attenuate infection or its resulting pathology, and nanovaccines that can enable alter vaccine delivery approaches and targeted delivery (Figures 1 and 2). Nanocarriers have been employed to target drug delivery for several other diseases, but can also be useful in reducing the side effects of current antiviral treatments. The development of smart strategies that can prevent or reduce viral transmission are top public health priorities due to their effectiveness in preventing disease outbreaks; significant effort has also focused on treatment options that can reduce the severity and time-course of infections when taken shortly after symptom onset or reduce the pathology of more severe disease. Oral antiviral drugs are emerging as a promising solution to control the severity of respiratory diseases when taken soon after symptoms onset (Figure 2), and multiple approaches are being employed to develop such drugs, but these may carry potentially significant risks or side effects. For example, the antiviral nucleoside analog drug molnupiravir developed by Merck substantially enhances the mutation rate during replication of the SARS-CoV-2 RNA genome to induce catastrophic viral replication errors that prevent further formation of functional virus, but the initial metabolite of this prodrug can also increase the mutagenesis rate in cultured cells.^53^ Another SARS-CoV-2 antiviral drug developed by Pfizer, paxlovid that contains nirmatrelvir and ritonavir. Nirmatrelvir functions as a peptidomimetic to inhibit the activity of the main SARS-CoV-2 protease nsp5 and has antiviral activity against coronaviruses known to infect humans.^54^ Ritonavir, a strong cytochrome P450 3A4 inhibitor, must be administered with nirmatrelvir to permit systemic nirmatrelvir concentrations to reach a therapeutic range, since cytochrome P450 3A4 is highly expressed in the liver and intestines. Due to its required inclusion of ritonavir, paxlovid is thus prone to interact with other coadministered drugs and may not be beneficial if a patient is receiving other medications for comorbid conditions.^55^ However, these detrimental effects could be minimized or avoided for current and future antiviral drugs—and other drug types—by employing targeted delivery approaches to selectively increase drug bioavailability only at the selected sites or tissues. One study has examined a nanoparticle-mediated strategy for targeted delivery of remdesivir, a nucleotide prodrug that promotes premature termination of viral RNA transcription^56^ and can reduce the risk of hospitalization and death in nonhospitalized patients at high risk for progression to severe disease.^57^ This study evaluated the potential for aerosol delivery of remdesivir-loaded liposome nanoparticles to enable direct drug delivery to affected respiratory tissue and avoid off-target effects that could arise during its standard, three-day intravenous systemic delivery protocol.^58^ Nanotechnology offers several nanocarrier platforms to permit selective and efficient drug delivery to targeted sites,^59−61^ which could increase drug bioavailability at therapeutic concentrations at sites of viral infections while reducing their systemic levels to decrease their systemic levels and associated off-target effects. Such nanocarriers can be employed to permit the delivery of multiple drugs to a target site to allow targeted delivery of synergistic drugs for more effective treatment.

Nanocarriers can be engineered to display factors that permit their targeted uptake by specific tissues or cell types that express the corresponding receptors, ligands, or other interacting factor.^62,63^ Further, due to their small size and other properties, these nanoparticles can readily penetrate most biological barriers, including the blood–brain barrier. This may be of significant importance since data now indicate that several established and emerging viral pathogens (e.g., human respiratory syncytial virus, influenza, and multiple coronaviruses, including SARS-CoV-2) exhibit neurovirulence, neuroinvasiveness, or neurotropism associated with neural pathology and thus the ability of drugs to cross the blood–brain barrier may be critical in treating the neuropathology of these infections. Notably, several nanomaterials (e.g., dendrimers, liposomes, carbon nanotubes, and polymeric nanoparticles) demonstrate great potential as nanocarriers for drug delivery across the blood–brain barrier,^64−67^ including functionalized nanoparticles that can be targeted for tissue- or cell-specific drug delivery.^68^ One caveat for this approach is that such nanoparticles are subject to clearance by the reticuloendothelial system and thus should be formulated to reduce their clearance by this mechanism.

Nanoparticles may also have effective therapeutic activity as nanodecoys that express receptors, ligands, or other materials that are specifically recognized by the target virus or factors that can have pathologic effects when expressed in excess during the response to infection. Respiratory virus exposure primarily occurs through direct contact with target receptors expressed by cells present on the epithelial surface of the nasal, oral, and tracheal mucosae. It may therefore be possible to employ biocompatible nanomaterials as virucidal agents or decoys in nasal or oral sprays to disrupt virion structure or block virus interaction with its target receptor to limit infection.^69,70^ This might also be useful early in infection or shortly after an exposure event if these approaches could reduce the burden of the active virus to attenuate virus replication and reduce the potential for virus transmission. For instance, SARS-CoV-2 employs angiotensin-converting enzyme 2 (ACE2), which is highly expressed in nasal and oral tissue, as its primary receptor for cell entry,^71,72^ and research has focused on the ability of nanodecoys that display ACE2 or cell membranes that highly express ACE2 to attenuate in vivo infections resulting from exposure to SARS-CoV-2 or an engineered SARS-CoV-2 pseudovirus.^70^ Severe respiratory virus infections, including severe COVID-19 cases, can produce dysregulated cytokine expression resulting in a cytokine storm and inducing acute lung injury that can progress to acute respiratory distress syndrome (ARDS) and multiple organ failure. Early intervention to reduce inflammation could reduce this progression to reduce ARDS morbidity and mortality resulting from severe respiratory virus infections, but care must be taken to avoid the deleterious effects of systemic immunosuppression. Targeted delivery of nanodecoys to the upper and lower respiratory tract using aerosol delivery approaches could, however, address this issue by restricting the immunosuppressive effect to the primary infection sites. Notably, one recent study employing a nanodecoy that carried high levels of ACE2 and multiple cytokine receptors found that this particle could both attenuate SARS-CoV-2 infection and independently reduce lung cytokine levels and injury stimulated by a nonviral stimulus.^69^ Studies have shown that extracellular vesicles derived from lung tissues and cells, and which carry viral receptors, are promising candidates for nanodecoys that can bind viruses to restrict their uptake by host cells.^73^ However, further work needs to be done to evaluate optimal approaches, doses, intervention times, and efficacy of such interventions. For instance, oral vaccine doses required to produce a robust immune response can be 100-fold higher than required in standard subcutaneous injection approaches,^74^ although this requirement might be attenuated by modifying the targeting, adjuvant, and other properties of these nanocarriers. Nasal delivery of nanocarrier-based vaccines could also reduce vaccine degradation and enhance its interactions with immune cells to decrease the required dosage while also promoting the development of a protective mucosal immune response.

## Conclusion

The recent history of multiple outbreaks by emerging coronaviruses (MERS-CoV and SAR-CoV) that culminated in the SARS-CoV-2-induced COVID-19 pandemic, and the constant threat of virus crossover events, has emphasized the need for multilevel approaches to combat future respiratory disease outbreaks capable of producing epidemics and pandemics. Current and future nanomaterial applications appear to hold great potential to prevent, detect, and treat existing and emerging respiratory infections to reduce their social and economic impact and avert future pandemics. One notable example of this potential was the rapid deployment of two major SARS-CoV-2 mRNA vaccines that used lipid nanoparticles as their delivery vehicles on the basis of previous studies. Research conducted during the COVID-19 pandemic has highlighted the potential of these approaches and the need for multidisciplinary research required to develop and validate future applications in key areas, including personal protection, environmental sanitation, and virus diagnosis and treatment. This perspective suggests areas where nanomaterial could contribute to improved applications, but it is to be expected that future research could greatly expand this list.

## Abbreviations

Severe acute respiratory syndrome coronavirus 2: SARS-CoV-2
COVID-19: coronavirus disease of 2019
VOC: variants of concern
MERS: middle east respiratory syndrome
DMA-PEGMA-QA: poly(dodecyl methacrylate)-poly(ethylene glycol) methacrylate-quaternary ammonium
RT-PCR: reverse transcription polymerase chain reaction
AI: artificial intelligence
IOMT: internet of medical things
ACE2: angiotensin-converting enzyme 2
ARDS: acute respiratory distress syndrome
